# Preclinical evaluation of antitumor activity of the proteasome inhibitor MLN2238 (ixazomib) in hepatocellular carcinoma cells

**DOI:** 10.1038/s41419-017-0195-0

**Published:** 2018-01-18

**Authors:** Giuseppa Augello, Martina Modica, Antonina Azzolina, Roberto Puleio, Giovanni Cassata, Maria Rita Emma, Caterina Di Sano, Antonella Cusimano, Giuseppe Montalto, Melchiorre Cervello

**Affiliations:** 1 Institute of Biomedicine and Molecular Immunology “Alberto Monroy”, National Research Council (CNR), Palermo, Italy; 2 Istituto Zooprofilattico Sperimentale della Sicilia “A. Mirri”, Histopathology and Immunohistochemistry Laboratory, Palermo, Italy; 3 Biomedical Department of Internal Medicine and Specialties, University of Palermo, Palermo, Italy

## Abstract

Hepatocellular carcinoma (HCC) is one of the common malignancies and is an increasingly important cause of cancer death worldwide. Surgery, chemotherapy, and radiation therapy extend the 5-year survival limit in HCC patients by only 6%. Therefore, there is a need to develop new therapeutic approaches for the treatment of this disease. The orally bioavailable proteasome inhibitor MLN2238 (ixazomib) has been demonstrated to have anticancer activity. In the present study, we investigated the preclinical therapeutic efficacy of MLN2238 in HCC cells through *in vitro* and *in vivo* models, and examined its molecular mechanisms of action. MLN2238 inhibited cell viability in human HCC cells HepG2, Hep3B, and SNU475 in a time- and dose-dependent manner. Flow cytometry analysis demonstrated that MLN2238 induced G2/M cell cycle arrest and cellular apoptosis in HCC cells. Cell cycle arrest was associated with increased expression levels of p21 and p27. MLN2238-induced apoptosis was confirmed by caspase-3/7 activation, PARP cleavage and caspase-dependent β-catenin degradation. In addition, MLN2238 activated ER stress genes in HCC cells and increased the expression of the stress-inducible gene *nuclear protein-1*. Furthermore, MLN2238 treatment induced upregulation of myeloid cell leukemia-1 (Mcl-1) protein, and *Mcl-1* knockdown sensitized HCC cells to MLN2238 treatment, suggesting the contribution of Mcl-1 expression to MLN2238 resistance. This result was also confirmed using the novel Mcl-1 small molecule inhibitor A1210477. Association of A1210477 and MLN2238 determined synergistic antitumor effects in HCC cells. Finally, *in vivo* orally administered MLN2238 suppressed tumor growth of Hep3B cells in xenograft models in nude mice. In conclusion, our results offer hope for a new therapeutic opportunity in the treatment of HCC patients.

## Introduction

Hepatocellular carcinoma (HCC) is known to be the second most frequent type of solid tumor^1^. Surgical intervention provides the best response in the early stages of the disease, but this approach is not feasible in all HCC patients. Standard therapy in advanced HCC patients involves the administration of Sorafenib, an oral multi-kinase inhibitor, which, unfortunately, has many side effects and increases life expectancy by only 3 months. This has led to the investigation of new treatment strategies and the identification of new target molecules, such as proteasome. Inhibition of proteasome causes an accumulation of misfolded proteins within the cell, an event that triggers the activation of the apoptotic pathway. Bortezomib (Velcade, PS-341), is a first-generation proteasome inhibitor, which the US Food and Drug Administration (FDA) has approved in multiple myeloma^2^ and non-Hodgkin’s lymphoma treatment^3^. At the molecular level, bortezomib treatment induces cell death through endoplasmic reticulum (ER) stress induction^4–7^, nuclear factor kappa B inhibition^8^, and caspase-8 activation^9^. However, although preclinical results have shown that bortezomib has antitumor effects in HCC^10–12^, a multicenter single-arm phase II trial conducted in cases of unresectable HCC showed that although bortezomib is well tolerated, it lacks significant activity^13^. Moreover, in many cases patients treated with bortezomib rapidly develop drug resistance, the mechanisms of which are poorly understood^14^.

The good clinical outcome observed with bortezomib in liquid tumor has led to the development of next-generation proteasome inhibitors to improve efficacy, avoid pharmaco-resistance and minimize cytotoxicity. Among them, MLN2238 (ixazomib) holds great promise: it is a next-generation reversible proteasome inhibitor, whose main value is that it can be administered orally. MLN2238 is the biologically active form of MLN9708 (ixazomib citrate), which in plasma or after exposure to aqueous solutions quickly hydrolyzes to MLN2238, the biologically active boronic acid. MLN2238 inhibits the 20 S proteasome chymotrypsin-like proteolytic (β5) subunit. It has a greater antitumor activity in solid and hematologic tumor models compared to bortezomib^15^. Several studies conducted in multiple myeloma patients have shown that ixazomib has great antitumor effects (NCT00963820; NCT00932698), and therefore the FDA has given its approval for treating this disease, also in association with other drugs, such as lenalidomide and dexamethasone (NCT02389517; NCT02917941)^16,17^. Furthermore, other newer reports have shown that MLN2238 is efficacious in other tumor cell types, such as osteosarcoma^18^, colon adenocarcinoma^19^, melanoma^20^, and neuroblastoma cells^21^. Treatment with MLN2238 results in the stabilization and accumulation of p21^Waf1/Cip1^^22^, E2F1 and p53^18^, which lead to the activation of caspase-3, -8, -9-dependent cell death pathways, with upregulation of Mcl-1 and NOXA^23,24^.

To date there are no studies on MLN2238 administration in HCC. In this study, we used HCC cells to explore the antitumor effects of MLN2238 *in vitro* as well as *in viv*o.

## Results

### MLN2238 reduces cell viability and proliferation HCC cells

**Fig. 1:**
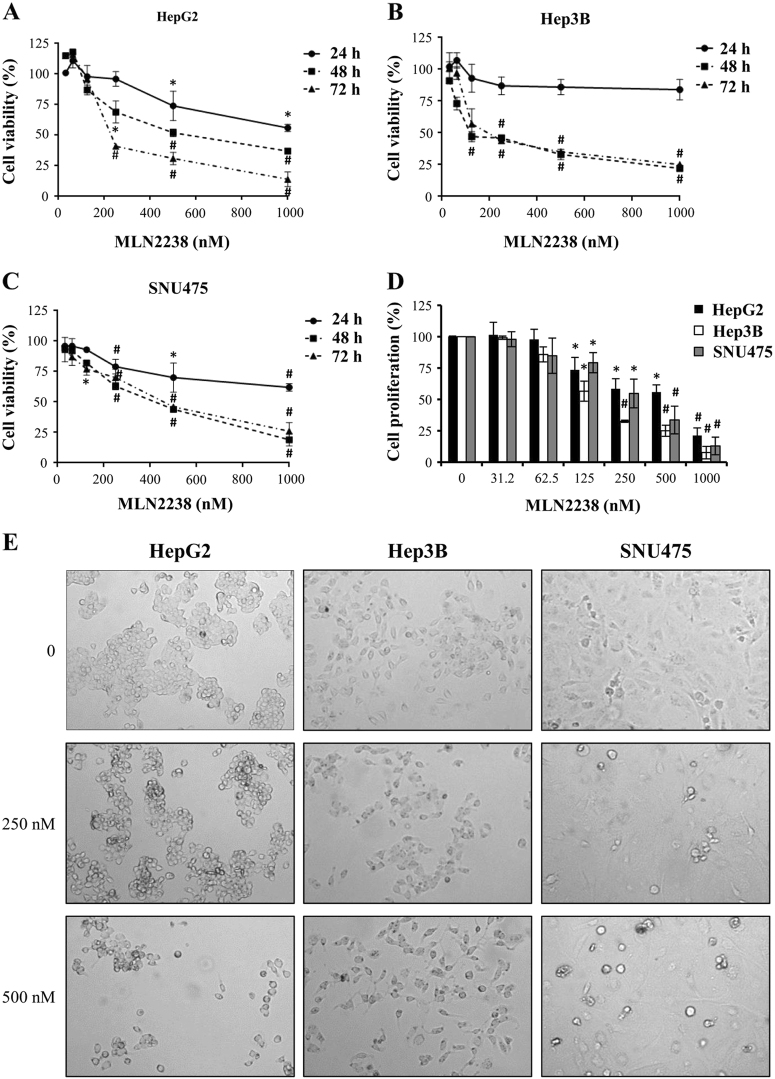
MLN2238 treatment alters cell viability, proliferation, and morphology in HCC cells. **a**–**c** Cell viability was evaluated by MTS assay. HepG2 **a**, Hep3B **b**, and SNU475 **c** cells were treated with increasing MLN2238 concentrations for 24, 48, and 72 h. **d** Proliferation was measured by BrdU incorporation into DNA. Cells underwent treatment for 48 h with increasing MLN2238 concentrations. **e** Morphological observations of MLN2238-treated cells by phase contrast microscope. Photographs were taken after 24 h of treatment (× 20 magnification). *P < 0.05 MLN2238 vs. control; ^**#**^*P* < 0.005 MLN2238 vs. control

The impact of MLN2238 on HCC cell viability was assessed by MTS assay. HCC cell lines HepG2, Hep3B and SNU475 were exposed to treatment for 24, 48, and 72 h with increasing concentrations of MLN2238. As shown in Fig. 1a–c, MLN2238 decreased cell viability depending on the dose and duration of treatment. The various cell lines exhibited differing degrees of sensitivity to MLN2238. At 24 h of treatment, HepG2 and SNU475 cells exhibited some sensitivity, but the effect did not reach the IC_50_ value. At 48 and 72 h, all cell lines showed sensitivity to MLN2238. As shown in Table 1, at 48 h, the IC_50_ value for Hep3B cells was 201 ± 31 nM, whereas for HepG2 and SNU475 cells values were 570 ± 22 nM and 387 ± 36 nM, respectively. At 72 h, the IC_50_ value for HepG2 cells was 281 ± 14 nM and the IC_50_ values for Hep3B and SNU475 were 260 ± 24 nM and 428 ± 81 nM, respectively.

**Table 1:** IC_50_ values for MLN2238 in HCC cells

	IC_50_ (nM, mean ± S.D.)		
	HepG2	Hep3B	SNU475
48 h	570 ± 22	201 ± 31	387 ± 36
72 h	281 ± 14	260 ± 24	428 ± 81

Furthermore, a BrdU incorporation assay was performed to determine cell proliferation after MLN2238 treatment. Fig. 1d shows that after 48 h MLN2238 significantly inhibited HCC cell proliferation according to the drug concentration.

In addition, inhibition of proliferation was evidenced by observations using optical microscopy. The images in Fig. 1e clearly show that the number of cells decreased at the various concentrations, and in addition, in HepG2 and SNU475 cells, a typical morphological feature of apoptotic cells was also observed, i.e, cells became rounded and detached from the substrate (Fig. 1e).

### MLN2238 induction of G2/M cell cycle arrest and p27 and p21 upregulation in HCC cells

**Fig. 2:**
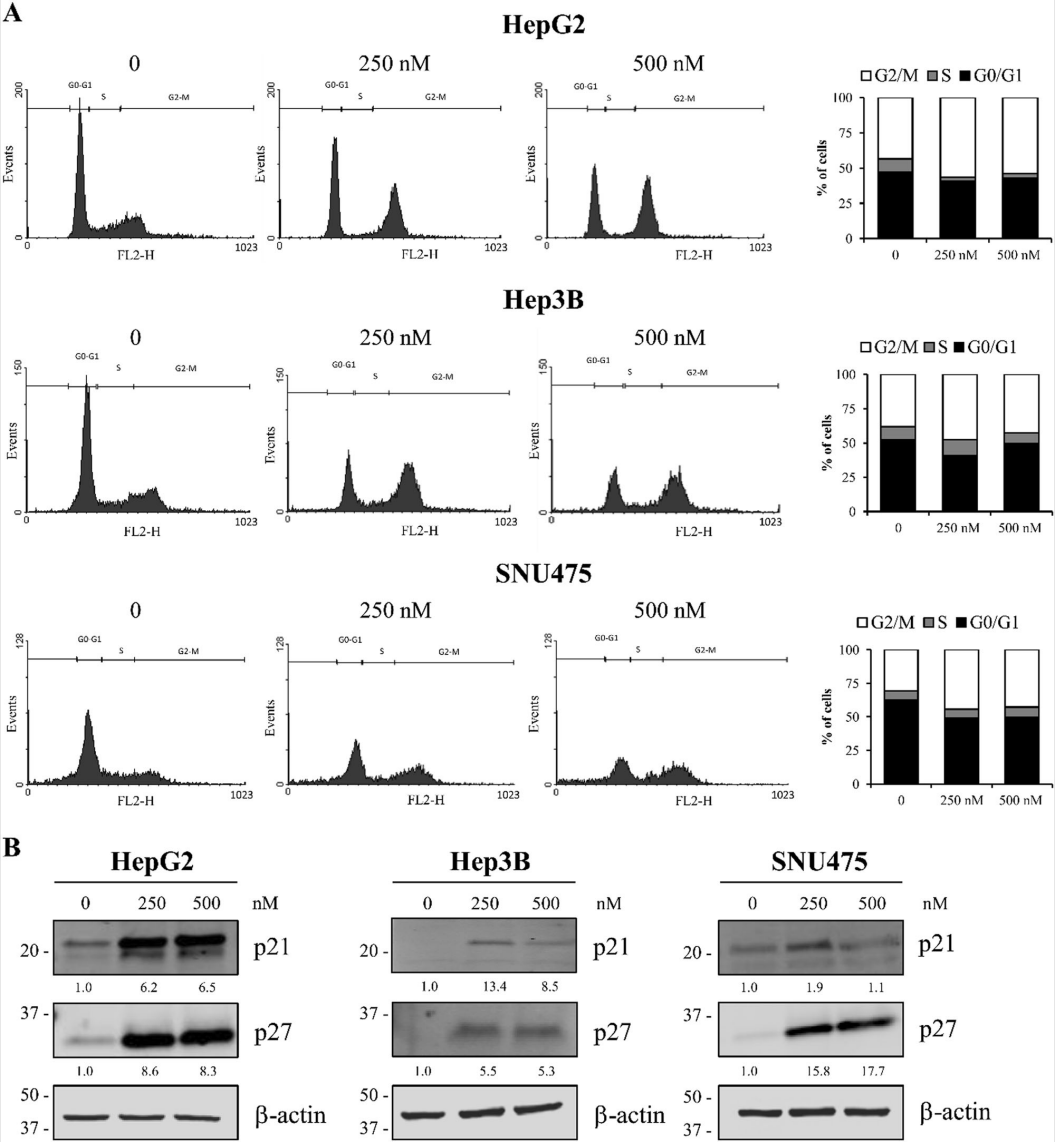
MLN2238 treatment induces cell cycle arrest and upregulated p21 and p27 expression in HCC cells. **a** Images representing cell cycle analysis in cells receiving 250 and 500 nM MLN2238 for 24 h. Left panels, flow cytometry was used to analyze the DNA content of cells after staining with propidium iodide. Right panels, summarized data of cell cycle distribution. Data are expressed as the percentage of cells in the G2/M, S and G0/G1 cell cycle phase, and are expressed as the means of three separate experiments. **b** Western blot analysis of p21 and p27 in HCC cells after treatment with 250 and 500 nM of MLN2238 for 24 h

To clarify MLN2238-induced inhibitory effects on HCC cell proliferation, the cell cycle phase distributions of HepG2, Hep3B, and SNU475 cells were examined by flow cytometry analysis. Fig. 2a shows representative cell cycle phase distributions of the controls and cells treated at 250 and 500 nM MLN2238 concentrations. Quantitative analysis (Fig. 2a, right side) showed that MLN2238 treatment for 24 h induced a higher cell population in the G2/M phase, and a lower population in the G0/G1 phase than in the control.

Next, to explore the molecular mechanism triggering MLN2238-induced cycle arrest in cells, the expression of proteins involved in controlling G2/M phase cell cycle progression was examined. Treatment with 250 and 500 nM MLN2238 increased expression of the inhibitors of cyclin-dependent kinase p21 and p27 (Fig. 2b).

### MLN2238 induces apoptotic cell death in HCC cells

**Fig. 3:**
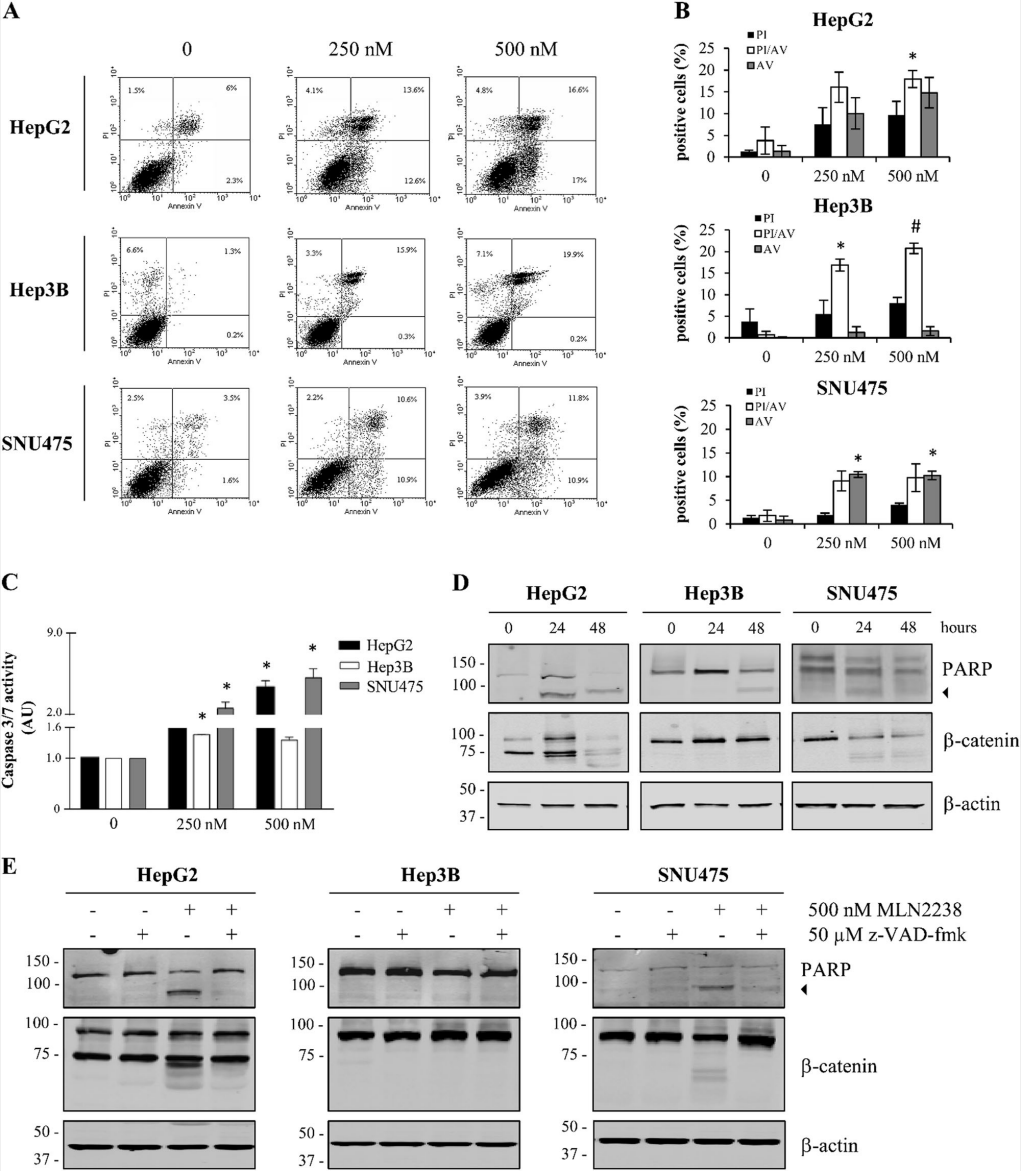
MLN2238 induces apoptosis in HCC cells. **a** Representative FITC-Annexin V/PI flow cytometry analysis of HepG2, Hep3B, and SNU475 cells after 24 h of incubation with 250 and 500 nM MLN2238. **b** Summarized data of apoptotic percentage distribution. **c** Caspase-3/7 activity levels in cells after treatment with 250 and 500 nM MLN2238 for 24 h. Data are given as AU normalized to control values and represent the mean ± S.D. (*n* = 3). **d** Representative western blotting of PARP and β-catenin in cell lines after treatment with 500 nM MLN2238 for 24 and 48 h. The 85 kDa form of PARP is indicated by an arrowhead. **e** Representative western blot analysis of PARP and β-catenin after treatment with 500 nM MLN2238 and/or 50 µM z-VAD-fmk for 24 h. The 85 kDa form of PARP is indicated by an arrowhead. *P < 0.05, MLN2238 vs. control; ^**#**^*P* < 0.005, MLN2238 vs. control

We further investigated whether MLN2238 elicited apoptotic cell death in HepG2, Hep3B, and SNU475 cells, using flow cytometry analysis. The percentages of early and late apoptotic and necrotic cells increased in comparison with the control according to the doses administered, suggesting that MLN2238 increased cell death in HCC cells (Fig. 3a, b).

Apoptosis induction was also investigated by measuring the activation of caspase-3/7 and analyzing Poly(ADP-ribose) polymerase (PARP) cleavage. Fig. 3 shows that at 24 h of treatment an increases in caspase-3/7 activity (Fig. 3c) in all cell lines was observed, whereas PARP cleavage (Fig. 3d) was observed only in HepG2 and SNU475 cells. However, in Hep3B cells PARP cleavage was observed after treatment for 48 h (Fig. 3d).

Proteasome inhibitors have been reported to activate apoptotic response through a specific caspase-dependent cleavage of β-catenin^25^. Therefore, we analyzed the expression of β-catenin following MLN2238 treatment. Fig. 3e shows that MLN2238 treatment triggered the cleavage of β-catenin in HepG2 and SNU475 cells. Furthermore, the involvement of caspases in PARP and β-catenin cleavage after treatment with MLN2238 was confirmed by using z-VAD-fmk, a cell-permeable and irreversible pan-caspase inhibitor. PARP and β-catenin cleavage was inhibited by co-treatment with z-VAD-fmk and MLN2238 (Fig. 3e).

The above findings suggest that MLN2238 induced the apoptosis pathway.

### MLN2238 induces ER stress

**Fig. 4:**
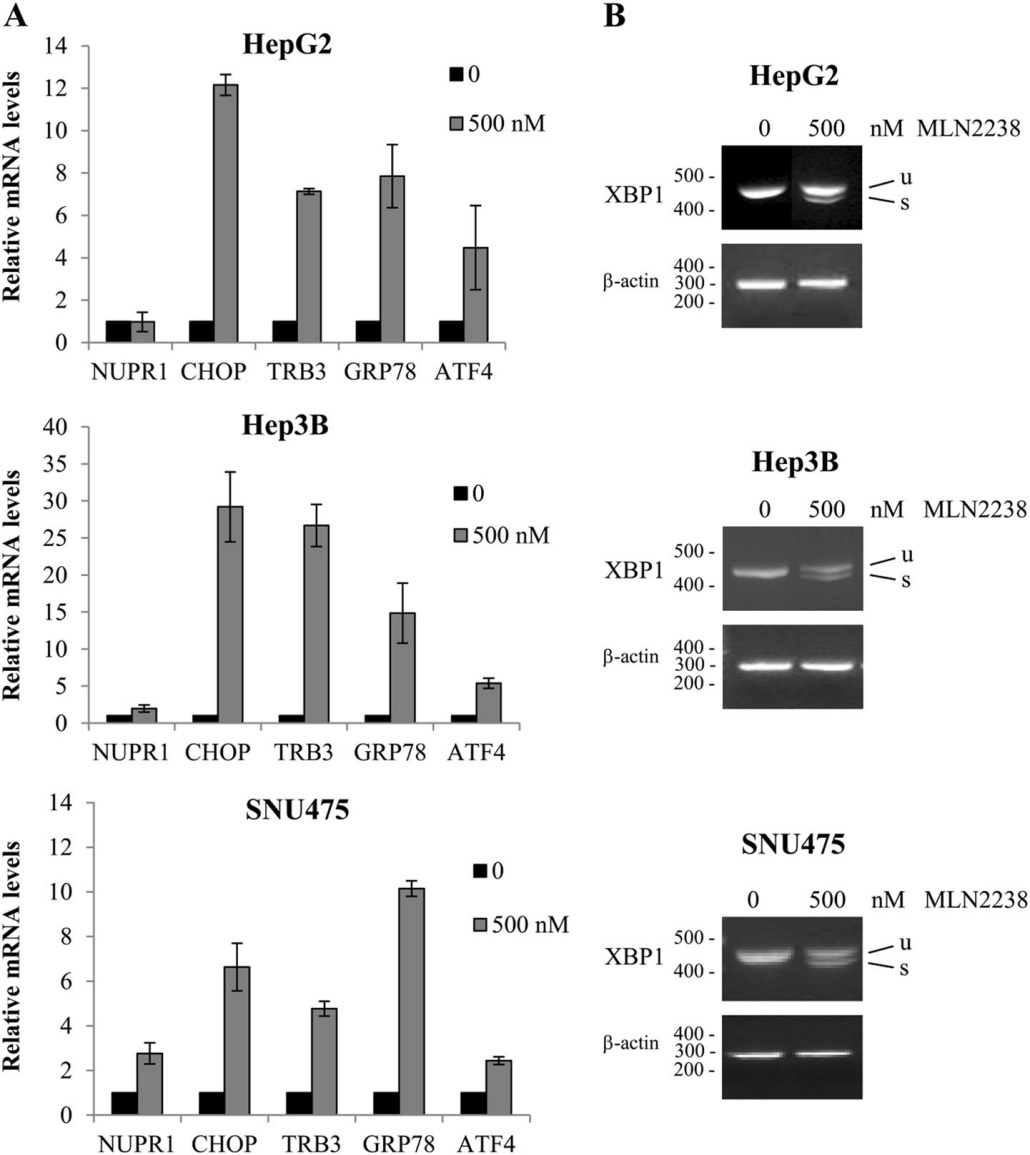
MLN2238 treatment induces ER stress in HCC cells. Effects of MLN2238 treatment with 500 nM of MLN2238 for 24 h on ER stress gene expression levels were determined by quantitative Real-Time PCR **a** and semiquantitative PCR **b**. **a** The relative gene expression was calculated (ratio of drug-treated samples vs. control) and corrected by the quantified level of β-actin expression. **b** Expression of XBP1 mRNA. *u* = unspliced XBP1 mRNA; *s* = spliced XBP1 mRNA

Several studies have established that proteasome inhibitors, including MLN2238, trigger the unfolded protein response and ER stress^4–7,26–28^. Therefore, we examined the effects of MLN2238 treatment on genes known to be regulated during ER stress response. As shown in Fig. 4, treatment with MLN2238 activated ER stress genes in HCC cells after 24 h. There was an upregulation of the ER stress-regulated genes, including *GRP78*, *ATF4*, *CHOP* and *TRB3* (Fig. 4a), and XBP1 mRNA splicing was also induced (Fig. 4b).

In human HCC tissues, we recently observed an overexpression of nuclear protein-1 (NUPR1), a stress-inducible protein involved in regulating cell survival, apoptosis, and invasiveness, as well as chemoresistance in HCC^29^. Therefore, expression levels of NUPR1 were also investigated after MLN2238 treatment. As shown in Fig. 4a, MLN2238 treatment increased NUPR1 mRNA expression in Hep3B and SNU475 cells after 24 h of treatment. These results confirm that MLN2238 treatment caused the activation of ER stress response in HCC cells.

### MLN2238 treatment induces Mcl-1 upregulation

**Fig. 5:**
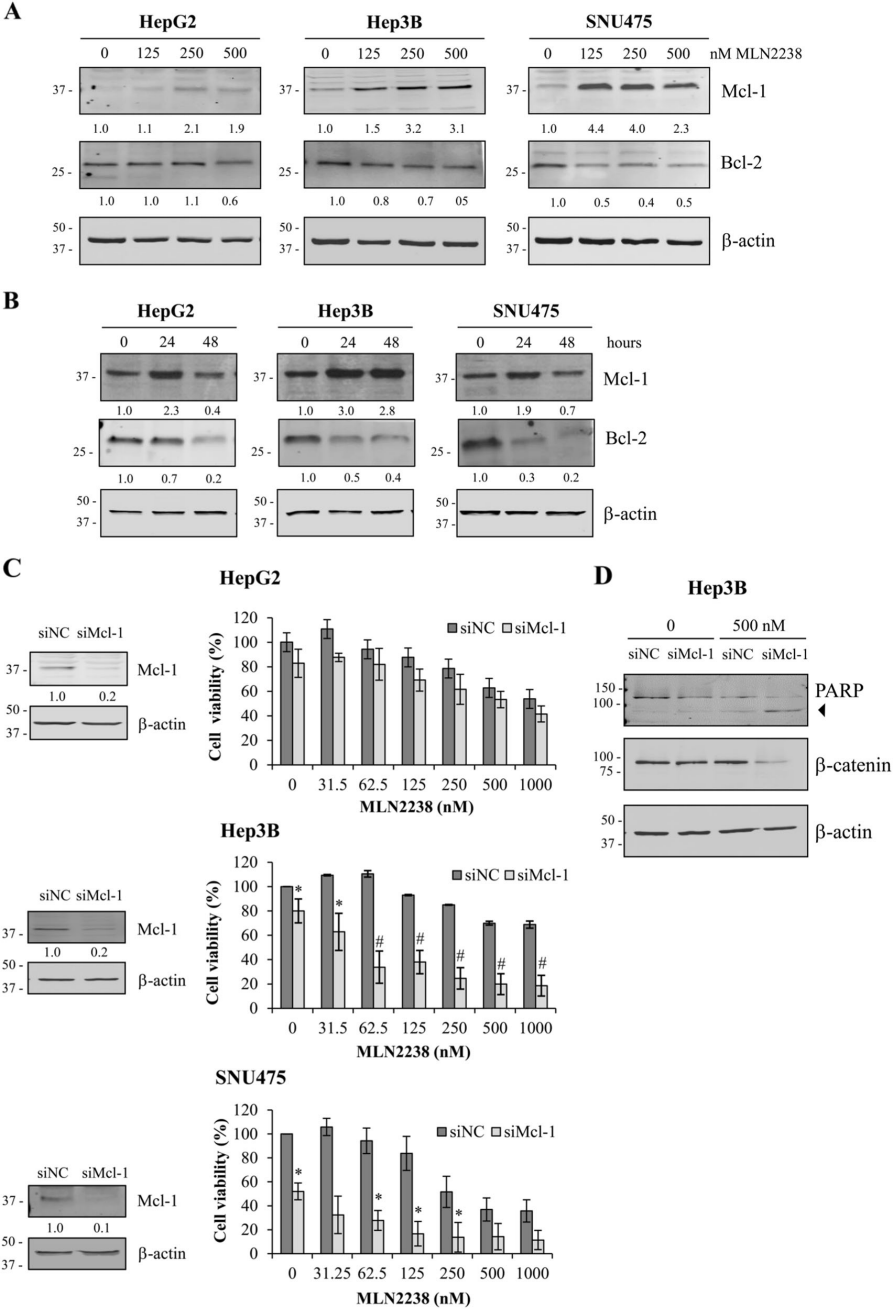
MLN2238 treatment regulates expression of anti-apoptotic proteins Mcl-1 and Bcl-2, and *Mcl-1* knockdown sensitizes HCC cells to MLN2238-mediated cell death. Dose- **a** and time-dependent **b** effects of MLN2238 treatment on Mcl-1 and Bcl-2 expression determined by western blot analysis. **a** Cells exposed to the specified MLN2238 concentrations for 24 h. **b** Cells treated with 500 nM of MLN2238 for 24 and 48 h. **c** Left panels, Mcl-1 expression levels after 72 h of transfection with Mcl-1 siRNA (siMcl-1) in cells compared with cells transfected with control siRNA (siNC). Right panels, viability of cells transfected with Mcl-1 siRNA (siMcl-1) after treatment with the indicated MLN2238 concentrations for 24 h. **d** Representative western blotting of PARP and β-catenin levels expressed in Hep3B cells transfected with Mcl-1 siRNA (siMcl-1) or control siRNA (siNC) after 24 h of treatment with 500 nM of MLN2238. The 85 kDa form of PARP is indicated by an arrowhead. *P < 0.05 MLN2238 vs. control; ^**#**^*P* < 0.005 MLN2238 vs. control

Numerous studies have demonstrated that proteasome inhibition can trigger the accumulation of myeloid cell leukemia-1 (Mcl-1), which is an anti-apoptotic protein belonging to the Bcl-2 family. To examine whether treatment with MLN2238 promotes the accumulation of Mcl-1 in HCC, HepG2, Hep3B, and SNU475 cells were exposed to 125, 250, and 500 nM of MLN2238 treatment for 24 h and with 500 nM for 24 and 48 h. Western blot analysis demonstrated that MLN2238 induced Mcl-1 increase in a dose-dependent manner in all HCC cell lines at 24 h (Fig. 5a), whereas at 48 h, the levels of Mcl-1 decreased in HepG2 and SNU475 cells, and remained higher than the control in Hep3B cells (Fig. 5b).

In addition, we evaluated whether treatment with MLN2238 induced the accumulation of other anti-apoptotic proteins. However, western blot analysis of Bcl-2 protein expression demonstrated that MLN2238 induced Bcl-2 downregulation according to the treatment dose and duration (Fig. 5a, b).

Therefore, to explore the relationship between the expression of Mcl-1 and cell susceptibility to MLN2238 treatment, HCC cells were transfected with Mcl-1-specific small interference RNA (siRNA) to inhibit *Mcl-1* gene expression. Transfection efficiency after 72 h of transfection with siMcl-1 was confirmed by a clear reduction in Mcl-1 protein expression levels (Fig. 5c, left side). Furthermore, MTS assays showed that *Mcl-1* knockdown (KD) significantly sensitized Hep3B and SNU475 cells to MLN2238 treatment compared with cells transfected with control siRNA (siNC), whereas although a reduction of cell viability was observed in HepG2 cells after *Mcl-1* KD, differences did not reach statistical significance (Fig. 5c, right side). In addition, in Hep3B cells silencing of *Mcl-1* promoted MLN2238-induced PARP cleavage and β-catenin reduction already at 24 h (Fig. 5d). Overall, these results suggest that on MNL2238 treatment, Mcl-1-induced expression protects HCC cells from MLN2238 antitumor effects.

### Co-treatment with MLN2238 and Mcl-1 inhibitor A1210477 synergistically reduces cell viability in HCC cells

**Fig. 6:**
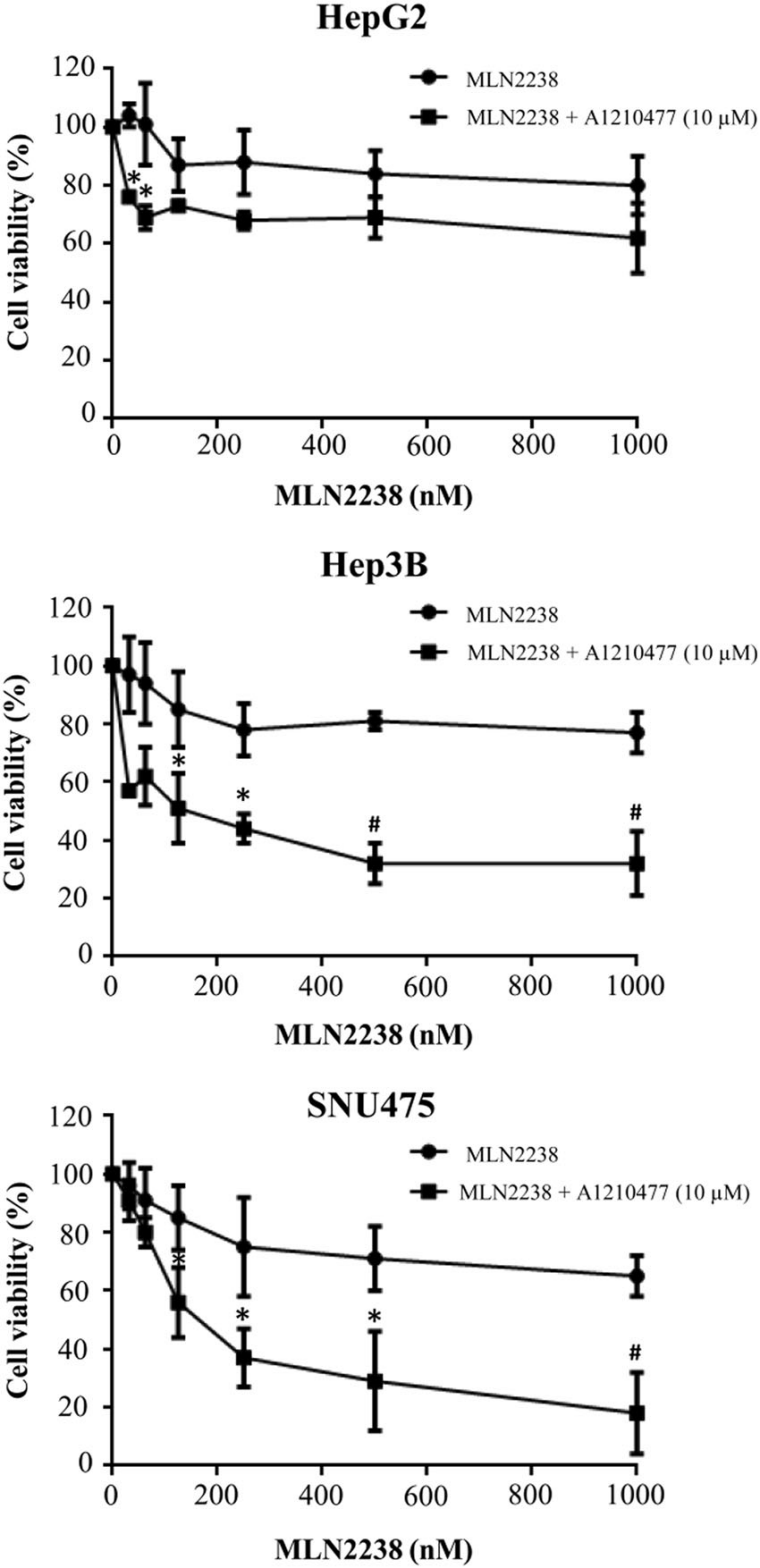
Treatment with MLN2238 in combination with A1210477 synergistically inhibits cell viability in HCC cells. Viability after treating HCC cells with different doses of MLN2238 + 10 µM A1210477 for 24 h was evaluated by MTS assay. *P < 0.05, 10 µM A1210477 + MLN2238 vs. 10 µM A1210477 alone; ^**#**^*P* < 0.005, 10 µM A1210477 + MLN2238 vs. 10 µM A1210477 alone

To explore the function played by Mcl-1 in MLN2238 resistance in HCC cells, we investigated whether the pharmacological inhibition of Mcl-1 with a potent and specific inhibitor, A1210477, would exert a similar effect to that obtained using the siRNA approach. We thus first determined the dose of A1210477 at which cell viability was minimally affected. At a dose of 10 μM A1210477 exerted only minimal effects on cell viability, with 90%, 85%, and 93% of cell survival in HepG2, Hep3B, and SNU475 cells, respectively (data not shown). Consequently, A1210477 alone (10 μM) or in combination with MLN2238 was used for cell treatment. Combinations of the two agents increased treatment efficacy compared with a single agent, as assessed by MTS assay at 24 h of treatment (Fig. 6). To define the type of interaction between the two agents, the combination index (CI) for each association was determined. As reported in Table 2, according to the CI, a combined treatment with different concentrations of MLN2238 and 10 μM A1210477 synergistically inhibited cell viability.

**Table 2:** MLN2238 in combination with 10 µM A1210477 elicited synergistic inhibition of cell viability in HCC cells after 24 h of treatment

	HepG2	Hep3B	SNU475
	A1210477	A1210477	A1210477
MLN2238 (nM)			
31.25	0.703	0.543	1.030
62.5	0.572	0.605	0.659
125	0.754	0.524	0.368
250	0.784	0.490	0.268
500	1.129	0.414	0.258
1000	1.453	0.456	0.209

The combination index (CI) values are indicated. CalcuSyn software was used to calculate the CI, where a CI < 1 indicated synergy,~1 indicated an additive effect and >1 indicated antagonism

### MLN2238 treatment inhibits *in vivo* tumor growth

**Fig. 7:**
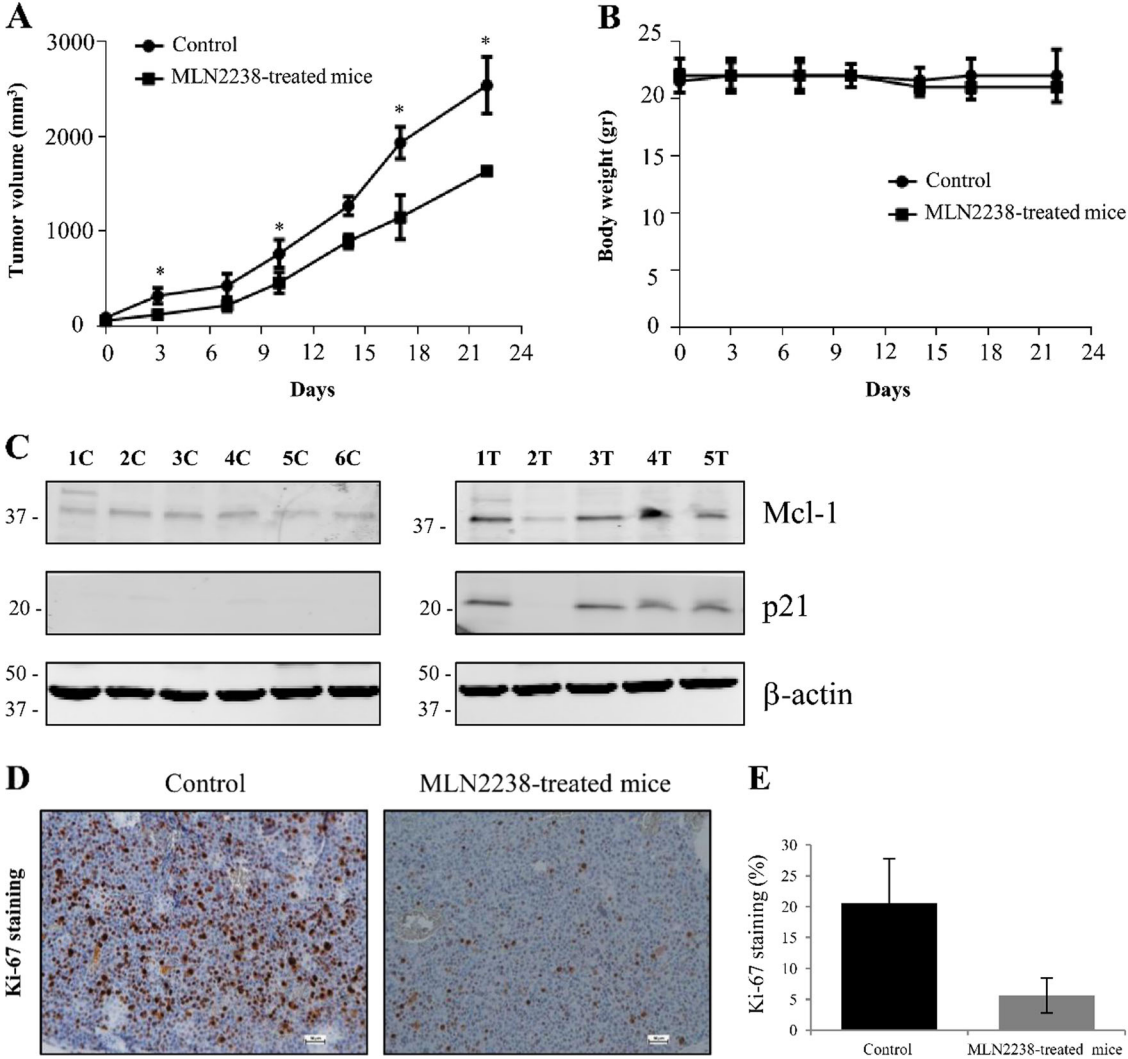
MLN2238 inhibits tumor growth *in vivo*. **a** Effect of MLN2238 on tumor development. A comparison was made between the tumor growth curve of MLN2238-treated mice (11 mg/kg) and that of control mice receiving only vehicle as treatment, *P < 0.05. **b** Analysis of body weight alterations. Animals were weighed two times/week and the data plotted in the graphs. **c** Representative immunoblotting of Mcl-1 and p21 expression levels in mice receiving only vehicle treatment (control: 1 C, 2 C, 3 C, 4 C, 5 C, and 6 C) and mice treated with MLN2238 (1 T, 2 T, 3 T, 4 T and 5 T). **d** Immunohistochemical staining of the Ki-67 proliferation marker in the control and MLN2238-treated mice (scale bar = 50 µm). **e** Data indicate the number of positive cells and are expressed as the means ± S.D. of five different fields in three sections of tumors from the control and the MLN2238-treated mice

A xenograft tumor model was utilized to demonstrate the antitumor efficacy of MLN2238 on HCC *in vivo*. Treatment with MLN2238 led to a significant reduction in tumor volume when compared with the volume measured in the control group (vehicle alone) (Fig. 7a). Furthermore, drug-associated cytotoxicity was monitored by analyzing changes in the body weight of animals. Treatment of mice with MLN2238 did not significantly modify body weight when compared to control group mice, which suggests that drug cytotoxicity was at a tolerable level (Fig. 7b).

To confirm the data observed *in vitro*, Mcl-1 and p21 expression in tumor tissues in mice receiving MLN2238 was investigated by western blot analysis (Fig. 7c). Mice treated with MLN2238 expressed higher levels of Mcl-1 and p21 than those of mice without the treatment. Therefore, these findings confirmed data previously observed in the *in vitro* experiments. In addition, histochemical analysis indicated that treatment with MLN2238 inhibited in tumor tissues the expression of Ki-67, a cell proliferation marker (Fig. 7d, e), confirming data obtained *in vitro* using a cell proliferation assay (bromodeoxyuridine (BrdU) assays).

## Discussion

Proteasome inhibitors represent a new class of promising therapeutic drugs in cancer^2,3^. However, although several preclinical studies have shown that proteasome inhibition by bortezomib could represent a viable therapeutic strategy for treatment of HCC^10–12^, clinical results obtained in patients with unresectable HCC have been disappointing^13^.

By contrast, the good clinical results observed with bortezomib in other tumor types have led to the development of next-generation proteasome inhibitors, such as MLN2238 (ixazomib)^15,22^, which has demonstrated better pharmacokinetics, pharmacodynamics, and antitumor activity compared with bortezomib^15,30^. Recently, ixazomib was shown to inhibit the *in vitro* growth of osteosarcoma^18^, colon adenocarcinoma^19^, melanoma^20^, and neuroblastoma cells^21^ *in vitro* and *in vivo*, using animal models.

Here, we demonstrated the potent antitumor effects of MNL2238 in HCC in both *in vitro* as well as *in vivo* models. To our knowledge, this is the first study assessing the use of the next-generation proteasome inhibitor MLN2238 in HCC.

Consistent with studies by other authors using different proteasome inhibitors, we showed that the cytotoxic effects of MLN2238-induced ER stress, apoptosis and cell cycle arrest at the G2/M phase, with upregulated p21 and p27 proteins^18–20,22,28^.

However, although the IC_50_ values of MLN2238 in Hep3B cells were lower than in the other two cell lines, the apoptotic response (in terms of PARP cleavage) to MLN2238 was delayed in time. These differences might be owing to different characteristics of differentiation, genetic defects, different ethnic origins, and to the biological behavior of these cell lines^25,31^, which are reflected in the tumor heterogeneity characterizing primary HCC. As an example: HepG2 cells carry a wild-type *p53* gene, Hep3B cells are *p53*-null and SNU475 cells carry the mutated *p53* gene. Interestingly, a recent study, by Dabiri et al.^32^, showed that *p53*-null colorectal cancer cells were transiently resistant, in the first 24 h, to the proteasome inhibitor bortezomib, whereas becoming responsive after 48 or 72 h of treatment, when the oncosuppressor isoform of p73, TAp73, translocated and accumulated in the nuclei of treated cells. On the other hand, KD of *p73* in *p53*-null cells significantly prolonged the time of resistance to bortezomib. These results suggest that TAp73 was responsible for the delayed pro-apoptotic effects of bortezomib in cells lacking functional p53^32^. Therefore, although we did not analyze the role of p73 during MLN2238 treatment in Hep3B cells, it could be hypothesized that a mechanism similar to those described by Dabiri et al., could explain the transient MLN2238 resistance observed in these cells. This interesting aspect needs further investigation and could be clarified by future studies.

We observed that the expression of Mcl-1, an anti-apoptotic protein, was upregulated upon treatment with MLN2238 in Hep3B cells after 24 h, and it also remained high at 48 h post-treatment, whereas expression declined at 48 h in the other two cell lines. It has been described that Mcl-1 accumulates as an undesired effect after exposure to proteasome inhibitors, and in addition, its expression decreases inhibitor efficacy^33,34^. Moreover, Mcl-1 expression continues in cells that have already undergone apoptotic response and is downregulated only at late time points after treatment^33^. This observation prompted us to assess the functional role of Mcl-1 in the apoptotic resistance to MLN2238 in Hep3B cells, especially considering that Mcl-1 has a pivotal role in HCC. Mcl-1 expression is higher in human HCC tissue than in the adjacent non-tumor liver^35,36^, and it also protects HCC cells against the drug-induced apoptosis of chemotherapeutic drugs^35,37^.

Here, we demonstrated that *Mcl-1* KD increased HCC cell sensitivity to MLN2238 and shortened the apoptotic response timing of HCC cells. In addition, we presented a novel strategy to enhance the efficacy of MLN2238 in HCC via modulation of Mcl-1 activity by pharmacological inhibition, using the small molecule Mcl-1 inhibitor A1210477. Our results demonstrate that combined treatment with MLN2238 and A1210477 represents a novel strategy for inducing synergistic inhibitory effects on cell viability in HCC cells. These data are consistent with results obtained by others using different proteasome inhibitors in various tumor types^33,38^, including MLN2238 ^39^.

Therefore, although our study demonstrated that MLN2238 is cytotoxic to HCC cells, potential mechanisms of chemoresistance should be taken into consideration, owing to Mcl-1 upregulation. However, as we have demonstrated here, this could be overcome by a combination strategy, using a pharmacological approach to inhibit Mcl-1 with small molecule inhibitors.

In conclusion, our findings suggest that proteasome inhibitors are promising drugs to tackle HCC and justify further investigation and clinical validation.

## Materials and methods

### Cell lines, cell culture, and reagents

The human HCC cell lines HepG2, Hep3B, and SNU475 were acquired from the American Type Culture Collection (ATCC) (HB-8065, HB-8064, and CRL-2236, respectively) and were maintained as previously described^40^. The cell lines were authenticated using short tandem repeat profiling (BMR Genomics, Padua, Italy). MLN2238, purchased from Selleck Chemicals (Houston, TX, USA), and A1210477, from Active Biochem (Bonn, Germany) were dissolved in dimethyl sulfoxide (DMSO).

### Cell viability and proliferation assays

A total of 3 × 10^3^ cells were added to 96-well plates and after 24 h were exposed to varying doses of MLN2238 alone, or combined with a subtoxic dose (10 µM) of Mcl-1 inhibitor A1210477. MTS assay was used to evaluate cell viability (see previous description)^41^. Data were recorded as a percentage of absorbance, comparing treated cells with controls (vehicle alone), and values expressed as the mean ± standard deviation (S.D.) of three experiments, each performed in triplicate.

To evaluate eventual effects of MLN2238 on cell proliferation, the incorporation of BrdU into DNA was measured by a colorimetric assay (Roche Diagnostics GmbH, Mannheim, Germany), as previously described^40^. Data are reported as the percentage of BrdU incorporation in treated cells compared with control cells. Values are reported as the mean ± S.D. of three experiments performed in triplicate.

### Cell cycle and apoptosis analyses by flow cytometry

HepG2 (6 × 10^5^), Hep3B (6 × 10^5^), and SNU475 (4 × 10^5^) cells were grown on 60-mm tissue culture dishes. At 24 h, the cells were exposed to MLN2238, and after 24 h flow cytometry was used for analyses (Becton Dickinson, Mountain View, CA, USA), performed as previously described^40^. The annexin V-FITC apoptosis kit (Dojindo Laboratories, Munich, Germany) was used to detect apoptosis, as previously described^42^. Results are presented as a percentage. The values indicated are the mean ± S.D. of two distinct experiments performed in triplicate.

### Caspase-3/7 activity assays

A total of 3 × 10^3^ HepG2, Hep3B, and SNU475 cells were distributed into 96-well plates. After 24 h, cells received 250 and 500 nM of MLN2238, and after an additional 24 h the caspase-3/7 activities were recorded with the Caspase-Glo 3/7 Assay (Promega), following the manufacturers’ instructions. Results are given as arbitrary units, indicating the mean ± S.D. of two separate experiments, each performed in duplicate.

### siRNA transfection

For siRNA transfection, 3 × 10^5^ Hep3B and HepG2 cells, and 1.8 × 10^5^ cells SNU475 cells were distributed into 6-well plates, containing culture medium without antibiotics. At 24 h, this was substituted with fresh culture medium and 50 nmol/l of Mcl-1 siRNA (siMcl-1) was used to transfect cells. Control cell transfection was performed with a Negative Control siRNA (siNC). Transfections were carried out with Lipofectamine RNAiMax (Invitrogen, Carlsbad, CA, USA), following the manufacturers’ instructions. siMcl-1 (SI02781205) and siNC (1027281) were acquired from QIAGEN (Germantown, MD, USA). After 24 h of transfection, cells were detached and seeded in 96-well plates or in 60-mm plates in preparation for MTS assay and for protein extraction, respectively.

### Western blotting

RIPA buffer (Cell Signaling Technologies Inc., Beverly, MA, USA) was used to obtain cell lysates, and western blotting was performed as reported previously^41^. Primary antibodies against β-actin were obtained from SIGMA (Milan, Italy), Mcl-1 was from Santa Cruz (Dallas, TX, USA) and PARP, p21Waf1/Cip1, p27 from Cell Signaling Technologies (Beverly, MA, USA), while β-catenin was from BD Transduction Laboratories (San Jose, CA, USA.). The relative expressions were calculated as the ratio of drug-treated samples vs. control (DMSO) and corrected using the quantified level of β-actin expression.

### RNA extraction, real-time, and semiquantitative PCR

Total RNA was extracted using the TRIzol method and 1 µg of RNA was used for reverse transcription to generate cDNA using the QuantiNova Reverse Transcription Kit (QIAGEN), following the instructions of the manufacturer.

Quantitative Real-time PCR was analyzed using the QuantiNova SYBR Green PCR Kit (QIAGEN) and QuantiTect primers specific for Mcl-1 (QT00094122), NUPR1 (QT00088382), CHOP (QT00082278), GRP78 (QT00096404), TRB3 (QT00088543), and β-actin (QT00095431). Data are expressed as the relative mRNA expression level of the different genes in treated cells compared with control cells. Values given are the mean ± S.D. of three different experiments performed in triplicate.

Semiquantitative PCR for the detection of XBP1 transcripts was performed using the following primers: 5′-CCTTGTAGTTGAGAACCAGG-3′ and 5′-GGGGCTTGGTATATATGTGG-3′ for XBP1 and 5′-CACCACACCTTCTACAATGAGC-3′ and 5′-GAGGATCTTCATGAGGTAGTCAGTC-3′ for β-actin, used as an internal control. The following programs were used to perform PCR reactions: 94 °C (5 min), then 94 °C (30 sec), 57 °C (30 sec) in the case of XBP1, and in the case of β-actin 60 °C (30 sec), then 72 °C (30 sec), and a final elongation step at 72 °C (10 min). Electrophoresis on agarose gel was performed to analyze amplified PCR products.

### *In vivo* studies

For the *in vivo* studies, MLN2238 was dissolved in 5% 2-hydroxypropyl-β-cyclodextrin at 1 mg/ml concentration. Female athymic nude mice (Fox1 nu/nu) 4 weeks of age were acquired (Envigo, Udine, Italy). Animals were left to acclimatize for 1 week. An inoculation of 10 × 10^6^ Hep3B cells in 200 µl of culture medium was made in the flank of the animal. When tumors reached the size of 150 mm^3^, the mice were randomly assigned to two groups of six animals each, receiving: 5% solution of 2-hydroxypropyl-β-cyclodextrin, two times/week by oral gavage in the control group (C) and 11 mg/kg of MLN2238, two times/week by oral gavage, in the treated MLN2238 group (T). Volumes of tumors and body weight were documented (see previous report)^41^.

After harvesting, each tumor was divided in half. One part was fixed in formalin and used for immunohistochemistry analysis, whereas the other was frozen in liquid nitrogen and stored at − 80 °C for western blotting. All the procedures were performed abiding by institutional guidelines, which comply with national and international laws and policies. Authorization for the study was obtained from the Italian Ministry of Health (no. 1187/2015-PR).

### Immunohistochemistry analysis

Immunohistochemical analysis was performed as reported previously^39^. To assess the proliferative index, nuclear antigen Ki-67 staining assay was carried out using a rabbit monoclonal antibody for Ki-67 (1:100) (Biocare Medical, CRM325). Expression level of Ki-67 was quantified as previously described^40^.

### Statistical analysis

Student’s *t*-test (two-tailed) was used for the statistical analysis. The statistical significance criterion was *p* < 0.05. To determine the synergistic effect, data analysis was performed using CalcuSyn Version 2.0 software (Biosoft, Cambridge, UK). CI <1 indicated synergy, ~1 an additive effect and >1 antagonism. IC_50_ was calculated by linear regression with the GraphPad Prism 6 software (GraphPad Software Inc., La Jolla, CA, USA).
